# Iatrogenic Iron Promotes Neurodegeneration and Activates Self-Protection of Neural Cells against Exogenous Iron Attacks

**DOI:** 10.1093/function/zqab003

**Published:** 2021-01-12

**Authors:** Maosheng Xia, Shanshan Liang, Shuai Li, Ming Ji, Beina Chen, Manman Zhang, Chengyi Dong, Binjie Chen, Wenliang Gong, Gehua Wen, Xiaoni Zhan, Dianjun Zhang, Xinyu Li, Yuefei Zhou, Dawei Guan, Alexei Verkhratsky, Baoman Li

**Affiliations:** 1 Practical Teaching Centre, School of Forensic Medicine, China Medical University, Shenyang, People’s Republic of China; 2 Department of Orthopaedics, The First Hospital, China Medical University, Shenyang, People’s Republic of China; 3 Department of Forensic Pathology, School of Forensic Medicine, China Medical University, Shenyang, China; 4 Faculty of Biology, Medicine and Health, The University of Manchester, Manchester, UK; 5 Achucarro Center for Neuroscience, IKERBASQUE, Basque Foundation for Science, 48011 Bilbao, Spain; 6 Department of Neurosciences, University of the Basque Country UPV/EHU and CIBERNED, Leioa, Spain; 7 Department of Forensic Analytical Toxicology, School of Forensic Medicine, China Medical University, Shenyang, China

**Keywords:** iron, metal implants, DMT1, astrocytes, Parkinson’s disease

## Abstract

Metal implants are used worldwide, with millions of nails, plates, and fixtures grafted during orthopedic surgeries. Iron is the most common element of these metal implants. As time passes, implants can be corroded and iron can be released. Ionized iron permeates the surrounding tissues and enters circulation; importantly, iron ions pass through the blood–brain barrier. Can iron from implants represent a risk factor for neurological diseases? This remains an unanswered question. In this study, we discovered that patients with metal implants delivered through orthopedic surgeries have higher incidence of Parkinson’s disease or ischemic stroke compared to patients who underwent similar surgeries but did not have implants. Concentration of serum iron and ferritin was increased in subjects with metal implants. In experiments in vivo, we found that injection of iron dextran selectively decreased the presence of divalent metal transporter 1 (DMT1) in neurons through increasing the expression of Ndfip1, which degrades DMT1 and does not exist in glial cells. At the same time, excess of iron increased expression of DMT1 in astrocytes and microglial cells and triggered reactive astrogliosis and microgliosis. Facing the attack of excess iron, glial cells act as neuroprotectors to accumulate more extracellular iron by upregulating DMT1, whereas neurons limit iron uptake through increasing DMT1 degradation. Cerebral accumulation of iron in animals is associated with impaired cognition, locomotion, and mood. Excess iron from surgical implants thus can affect neural cells and may be regarded as a risk factor for neurodegeneration.

## Graphical Abstract

**Graphical Abstract:**
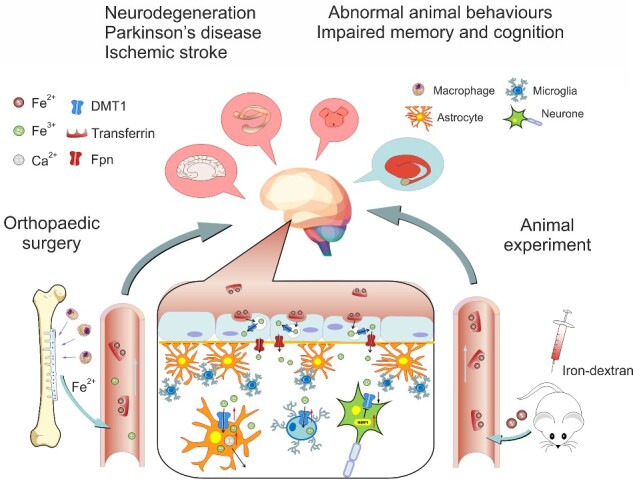

## Introduction

Every year, millions of metal implants such as metal nails, metal plates, and other fixtures are used for orthopedic intervention. At present, the follow-up mainly focuses on the operative prognosis, functional recovery, complications, and secondary pathologies. Effects of metal implants on the nervous system and neurological diseases, however, are rarely considered. Metal implants are mainly composed of pure iron or iron-based alloys, the latter being more widely adopted in clinical practice. The common metal alloys contain Mn, Ti, Mg, Al, Co, Si, etc., yet iron remains the primary element.^1^ Iron-based implants in the body can be corroded and iron in the form of ferric or ferrous (Fe^3+^ or Fe^2+^) may permeate in the surrounding tissues and enter circulation; of note Fe^3+^ and Fe^2+^ can be mutually converted by ferroxidase or ferrireductase.^2–4^ Corrosion of metallic components in the tissues may trigger inflammation and accumulation of iron in macrophages in months or years following the implantation.^3^^,^^4^ Manipulations with iron content in rodents through the diet, intraperitoneal (i.p.) injection, or subcutaneous infusion results in iron deposition in different tissues and in plasma.^5^

Ionized iron crosses blood–brain barrier (BBB) and blood–cerebrospinal barrier.^6^ Transport of iron across the BBB is mediated either by transferrin receptor-mediated internalization of Fe^3+^-bound to transferrin (holo-TF), or, for non-TF-bound iron, by membrane transport of Fe^2+^ mediated by divalent metal ion transporter 1 (DMT1/SLC11A2), which underlies Fe^2+^ uptake through the plasma membrane or from endosomes.^7^^,^^8^ Aberrant iron accumulation in the brain has been reported to be associated with neurodegenerative diseases, such as Alzheimer’s disease (AD), Parkinson’s disease (PD), and Huntington disease.^9–11^ Iron is associated with production of reactive oxygen species (ROS), which injure cellular membranes, proteins, and deoxyribonucleic acid.^12^ Oxidative stress resulting from the accumulated iron triggers neuronal death leading to neurodegeneration.^13^ The DMT1 is an H^+^-driven multimetal transporter responsible for transmembrane transport of nontransferrin-bound iron.^14^ In the central nervous system (CNS), DMT1 is widely expressed in neurons, astrocytes, microglial cells, endothelial cells, and oligodendrocyte progenitors,^15^ although how iron affects distinct neural cell types in vivo remains mostly unknown.

In this study, we performed retrospective analyses of patients with the history of metal implants; in particular, we focused on patients diagnosed with PD, ischemic stroke, or neurologic tumors (a disease-related control group). We discovered that metal implantation increased the occurrence of PD and ischemic stroke. Subsequently, we compared the PD occurrence in the patients subjected to orthopedic surgeries with and without metal implants. In addition, we measured the levels of serum iron, ferritin, and transferrin in the healthy control subjects and in patients who underwent surgical removal of implants immediately prior to the surgery. In the animal model, we studied how exogenous iron acts on neural cells, and we found the opposite effects of iron on the levels of DMT1 in neurons and glia. Glial cells respond to exogenous iron attack by upregulating DMT1 expression, whereas neurons accelerated DMT1 degradation; both these processes being arguably neuroprotective. Our study suggests that iron intake associated with orthopedic implants should be considered as a risk factor for neurological disorders.

## Materials and Methods

### Population Analysis

First, we set up a case-control study with common cerebral diseases, including 500 patients diagnosed with PD, 500 cases of ischemic stroke, 200 cases of brain tumor (glioma, meningioma, and hypophysoma), and 700 healthy controls. Patients with tumors were used as the disease-related control group. Then, for investigating the effects of orthopedic surgeries with and without metal implants on the risk of PD, we established a case-cohort study with the inpatients from orthopedic departments, including 15 000 patients with metal implant surgeries and 7500 patients who underwent orthopedic surgeries without implantation. To further analyze the effects of metal implants, blood samples were collected from 50 inpatients before surgical removal of metal implants and 50 healthy controls, all these participants gave their written informed consent to take part in this study. Both groups have the similar average age and the same gender ratio, 30 male and 20 female. The average holding time of metal implants before removal surgery was 18.98 ± 2.40 months (*n* = 50), and these metal implants were iron-based alloys grafted to patients with fracture of femoral shaft. The healthy subjects were the age-matched individuals who did not have neurological diseases and the history of surgeries. The anamneses were collected from the First Affiliated Hospital of China Medical University, the biggest general public hospital in the northeast of China with about 3 million outpatients and 159 000 inpatients every year. We selected cases of inpatients with definitive diagnosis from 2009 to 2018. Population sources were from the four provinces of China (Heilongjiang, Jilin, Liaoning, and Neimenggu). The individuals in healthy control group were similarly selected from these four provinces. This study was authorized and approved by Medical Ethics Committee of China Medical University, No. [2019]059.

### Data Collection

For the study of the link between metal implants and PD, stroke, or brain tumor, the collected data included age, gender, race, educational background, contact address, and the history of iron-based alloys implants. Among these factors, age and the time from metal implant surgery to be diagnosed related disease were quantified. The inclusion criteria were (1) ages more than 18 years, (2) PD diagnosis: patients were diagnosed according to UK PD Society Brain Bank Clinical Diagnostic,^16^ (3) ischemic stroke—diagnosed by CT or MRI images, (4) brain tumor—identified by histological analysis of tissues removed from neurosurgery excision. Healthy controls were selected from age-matched individuals, who did not have neurodegenerative diseases, stroke, brain tumor, and other cerebral diseases. For all groupings, the exclusion criteria were (1) the patients with any psychiatric diseases or with two or more cerebral diseases together; (2) the patients diagnosed with cerebral diseases before the orthopedic surgeries; (3) the patients with the history of any other surgery except the study-relevant orthopedic surgeries or neurosurgeries; (4) the patients whose contact were lost or who had difficulties in language communication.

### The Observation of Removed Metal Plates

Under the permission of patients and the management regulations of operating rooms, metal implants removed from the patients were inspected and images of the surface were taken by the video microscope (SC-HM1001, Chensheng Optical Instruments Co., Ltd, Shenzhen, China).

### Laboratory Determinations

Serum iron was measured by Sysmex xs-800i Automatic Hematology Analyzer (SYSMEX Corp., Kobe, Japan). Serum ferritin was detected with electrochemiluminescence technology and measured by Roche ELECSYS 2010 analyzer (Roche Diagnostics Ltd., Basel, Switzerland). Transferrin was determined with an immunonephelometric assay and a SIEMENS BN^TM^ II fully automatic analyzer (Siemens Healthcare Diagnostics Inc., Shanghai, China).

## Animals

Male wild type mice, as well as B6.Cg-Tg(Thy1-YFP)HJrs/J and FVB/N-Tg(GFAP-GFP)14Mes/J strains were obtained from Jackson Laboratory (Bar Harbor, ME, USA). Mice were 10–12 weeks old (∼25 g) and were kept in standard housing conditions (22°C ± 1°C; light/dark cycle of 12 h/12 h) with food and water available ad libitum. All operations were carried out in accordance with the USA National Institutes of Health Guide for the Care and Use of Laboratory Animals (NIH Publication No. 8023) and its 1978 revision, and all experimental protocols were registered in and approved by the Institutional Animal Care and Use Committee of China Medical University.

## Materials

Most chemicals, including iron dextran (ferric hydroxide dextran complex; Cat#: D8517) and 4′, 6-diamidine-2′-phenylinedole dihydrochloride (DAPI; Cat#: D3571) were purchased from Sigma (MO, USA). The primary antibody raised against DMT1 (Cat#: sc-166884) was purchased from Santa Cruz Biotechnology (CA, USA). Primary antibody raised against NeuN (Cat#: PA5-78639), GFAP (Cat#: PA5-78639) and secondary antibodies Alexa Fluor^TM^ 555 donkey anti-Rabbit (Cat#: A31572) and Alexa Fluor^TM^ 488 donkey antimouse (Cat#: A21202) was purchased from Thermo Fisher Scientific (CA, USA). Primary antibody raised against Iba-1 (Cat#: 019-19741) was purchased from Wako Pure Chemical Industries (Osaka, Japan). Micro serum iron concentration assay kit (Cat#: BC1735), potassium ferrocyanide, and diaminobenzidine (DAB) kit (Cat#: DA1015) were purchased from Solarbio Life Sciences (Beijing, China). TUNEL apoptosis detection kit (Cat#: 11772457001) was purchased from Roche-Sigma (USA). The ROS assay kit (Cat#: BB-470532) was purchased from BestBio (Shanghai, China). The quantitative PCR (qPCR; Cat#: RR820A and RR047A) was purchased from TaKaRa Bio (Kusatsu, Japanese).

## Iron Treatments

Iron dextran was dissolved in water at the weight ratio of 1:3 (iron/dextran), the solution of iron dextran was hypodermically injected 200 µL with iron content 1, 2, or 4 mg/kg/day for 6 days, once every day, the same concentration of dextran without iron in the same volume was injected to the control group.

## Immunohistochemistry

Brain tissue was immersed in 4% paraformaldehyde and cut into 50 μm slices. As described previously,^17^^,^^18^ slices were preincubated with normal donkey serum (NDS, 1:20; Jackson Immuno-Research Laboratory) for 1 h and a mixture of primary antibodies overnight, mouse anti-DMT1 (1:100), rabbit anti-NeuN (1:200), rabbit anti-GFAP (1:200), or rabbit anti-Iba1 (1:200). After several rinses, the slices were incubated with Alexa fluor-conjugated secondary antibodies for 2 h at room temperature, DAPI (1:1000) was used to identify cell nuclei. Immunofluorescence was imaged using a confocal scanning microscope (DMi8, Leica, Germany). To measure the level of DMT1 in neurons, astrocytes, and microglia, we calculated the mean intensity of DMT1 immunofluorescence which was colabeled with NeuN, GFAP, or Iba1 in cortical, hippocampus (HP), substantia nigra (SN), and striatum (ST) regions. The background intensity of each image was collected in cell-free parenchyma in the same field of view and subtracted from the immunofluorescence intensity. The intensity of DMT1 immunofluorescence from each group was normalized to the intensity measured from the control group.

### Perl’s Staining

Tissue iron accumulation was visualized by Perl’s staining with modifications. The slices were immersed in the mixed reaction liquid (4% potassium ferrocyanide: 4% hydrochloric acid = 1:1) at 37°C for 30 min and then stained using a DAB kit for 4 min. The slices were imaged using an optical microscope (BX53, Olympus, Japan or Carl Zeiss Promenade 10, Jena, Germany). The iron accumulation was calculated by the ratio of the Perl’s stained area and the total area.

### Animal Serum Iron Concentration

The blood samples were centrifuged at 3000 r/min for 20 min to collect the supernatant. The concentration of serum iron was measured with an assay kit and the operations were followed the manufacturer’s protocols. The absorption at 520 nm was measured using ELX808 microplate reader (BioTek Instruments Inc., VT, USA). The iron concentration was calculated by the curve of standard substance.

### Sorting Neural Cells through Fluorescence-Activated Cell Sorter

To measure the mRNA of DMT1 and Ndfip1, we sorted the cells labeled by fluorescent markers YFP for neurons (Thy1-YFP transgenic mice) and GFP for astrocytes (GFAP-GFP transgenic mice). As described previously,^19^^,^^20^ for sorting the microglia the cells isolated from wild-type mice were labeled with Iba1 antibody. The labeled cells were sorted with fluorescence-activated cell sorting (FACS) technology. Dead cells were excluded based on positive DAPI signals. The cells were sorted in cold Minimum Essential Media (Cat#: 11095080, Gibcao-Thermo Fisher Scientific, CA, USA) containing 1% Bovine Serum Albumin (Cat#: FA016, Genview, TX, USA). The RNA of the sorted cells was extracted by Trizol (Cat#:T9424, Sigma-Aldrich, MO, USA).

### The qPCR

Total RNA was reverse transcribed and PCR amplification was performed in a Robo-cycler thermocycler, as our previous description.^18^^,^^19^ Briefly, relative quantity of transcripts was assessed using 5-folds serial dilutions of RT product (200 ng). RNA quantity was normalized to glyceraldehyde 3-phosphate dehydrogenase (GAPDH) before calculating relative expression of DMT1 or Ndfip1. Values were expressed as the ratio of the relative expression of DMT1/GAPDH or Ndfip1/GAPDH.

## Ethogram

As described previously,^21^ we assessed the mice status according to general appearance parameters (GAP) assessments. A score of 0 or 1 for the categories of activity, posture, breathing pattern, coat condition, and interaction with other mice was given.

### Morris Water Maze Test

As in our previous description,^18^ the Morris water maze test was carried to characterize spatial learning and memory performance. Mice were trained with four trials for five consecutive days daily. Over the period, mice were conducted to locate and escape onto the platform. The platform position was fixed throughout the test. Each mouse was conducted to swim from a different starting quadrant. Mice failing to swim to the target position within 90 s were guided to the platform and allowed to remain on it for 30 s. In the memory retention session, the hidden platform was removed, the mice were given a time of 60 s to probe, and the time spent in the target quadrant by each mouse was collected.

### Pole Test

The pole test for assessing movement abnormalities was performed as previously described.^18^ A vertical rough-surfaced pole (diameter 1 cm; height 55 cm) was used in this test and the mouse was placed down-upward on its top. The movement time from the pole top to the floor (T-LA time) was measured. The total time was measured with a maximum duration of 30 s.

### Rotarod Test

To assess motor coordination in mice, a rotarod test was performed as previously described.^18^ Mice were placed on a rotating bar with a rotation speed of 18 rpm. The time on the rotating bar was recorded as the latent period. Latency to fall was recorded with a maximum of 60 s. Each mouse was given twice consecutive trials and mean value was used for analysis.

### Open Field Test

Open field test for assessing anxiety behavior was conducted with an open field box (60 × 60 × 40 cm) which was divided into 9 squares, as previously described.^18^ Each mouse was placed in the center square. Behaviors were recorded for 6 min. The parameters included total traveled distance and time spent in the central area was used for analysis.

### TUNEL Staining and Analysis

To evaluate neuronal apoptosis in four different regions, TdT-mediated dUTP-biotin nick end labeling (TUNEL) in conjunction with immunofluorescent staining for NeuN was performed as previously described.^17^ Briefly, TUNEL staining was performed using an in situ cell death detection kit, following the manufacturer’s protocol. The brain slices were incubated overnight at 4°C with anti-NeuN antibody (1:100) followed by incubation with secondary antibody (1:400) for 2 h at room temperature. Finally, the slices were stained with DAPI (1:2000). The images were taken using a confocal microscope (DMi8, Leica, Germany) by an investigator blinded to the experimental design. Similar cerebral regions from six mice in every experimental group were calculated, and the average percentage of TUNEL+/Total DAPI+ was statistically analyzed.

### Detection of ROS

The tissues from frontal cortex (FC), HP, SN, and ST were divided and centrifuged ×1000 g at 4°C for 10 min. The supernatant was remained and the protein concentration was determined. As per the manufacturer’s protocols of ROS assay kit, adding 10 µL of supernatant and 190 µL of BBoxi Probe O12 detector to a 96-well plate, which was incubated for 30 min at 37°C in dark. Then fluorescence intensity was measured by fluorescence microplate reader (Infinte M200 Pro, Tecan, Switzerland). The detection result was presented as the ratio of fluorescence value/mg protein.

## Statistical Analysis

We used GraphPad Prism 5 software (GraphPad Software Inc., La Jolla, CA) and SPSS 24 software (International Business Machines Corp., NY, USA) for the data statistical analysis, the level of significance was set at *P* < 0.05. For the case-control study, unpaired two-tailed Student’s *t*-test and *X*^2^ test or Fisher exact test (when expected frequencies of the cells of a contingency table <5 were over 20%) were used for analyzing different kinds of data, respectively. For animal experiments, analysis of variance followed by Fisher’s least significant difference or a Tukey–Kramer post hoc multiple comparison test for unequal replications was used for statistical analysis.

## Data Availability

The data that support the findings of this study are available from the corresponding author upon reasonable request.

## Results

### High Incidence of Metal Implantation in PD and Ischemic Stroke

Table 1 shows the general characteristics of the subjects under study. There was no significant difference in age-distribution between control group and any of disease groups (Figure S1A). Similarly, there was no gender-distribution difference, and the male–female ratio was close to 1.0 in all groups. There was no difference in educational level and ethnical origin (individuals of Han ethnical origin accounted for more than 96.43% in control or any case group, which was matched to the national population percentage of Han ethnicity in China). The incidence of metal inserts was 3.60%, *X*^2^(1)=8.307, *P* = 0.004 in PD group, and 5.00% (*X*^2^(1)=16.225, *P* < 0.0001) in ischemic stroke group, while it was only 1.14% in healthy control group. In the cerebral tumor group, the insert incidence was 1.50% (*P* = 0.716) which was not significantly different from healthy control group.

**Table 1.** General Characteristics of 500 PD Cases, 500 Cerebral Ischemia Cases, 200 CNS-Tumor Cases, and 700 Healthy Controls

Feature	Healthy control	PD	Cerebral ischemia	CNS tumor
Total number	700	500	500	200
Age (years)	66.95 ± 11.29	67.70 ± 10.29 *t*(1198)=1.184; *P* = 0.237	67.75 ± 10.56 *t*(1198)=1.244; *P* = 0.214	65.36 ± 10.54 *t*(898)=1.772; *P* = 0.077
Gender				
Male	359	250	256	103
Female	341	250 *X*^2^ (1)=0.193; *P* = 0.661	244 *X*^2^ (1)=0.001; *P* = 0.977	97 *X*^2^ (1)=0.003; *P* = 0.957
Education level				
Primary and secondary education	474	345	325	133
Skilled vocation and high school education	142	93	113	36
University and postgraduate education	84	62 *X*^2^ (2)=0.532; *P* = 0.766	62 *X*^2^ (2)=1.096; *P* = 0.578	31 *X*^2^ (2)=1.936; *P* = 0.380
Ancestry				
Han ethnical origin	675	497	495	194
Non-Han ethnical origin	25	3	5	6
Metal inserts history	**8**	**18**	**25**	**3**
Metal inserts ratio (%)	**1.14**	**3.60**	**5.00**	**1.50**
Test statistic		*X* ^2^ (1)=8.307; *P* = 0.004 *	*X* ^2^ (1)=16.225; *P* = 0.000056 *	***P* = 0.716**

Bold values represents the positive numbers and ratios of subjects having metal inserts history.

*Signicifant difference from healthy control group.

Detailed distribution of clinical cases with inserts was further analyzed with the results shown in Table S1. In the control group, 8 out of 700 individuals had metal implants history; in PD group 18 subjects out of 500 had metal inserts, whereas in ischemia group 25 patients out of 500 had metal implants. According to the age at the moment of diagnosis, all subjects were divided into four age groups, <46, 46–60, 61–75, and >75 years. Compared to the age distribution in the control group, the metal insert cases were more frequent in the last two groups (Figure S1B). In PD group, 11 out of 244 patients from 61 to 75 years old group had inserts (*X*^2^(1)=4.414, *P* = 0.036), in patients older than 75 years, 3 out of 38 underwent metal implantation (*P* = 0.043). In ischemia group, the inserts frequencies were significantly different from healthy subjects in three age groups: 5 out of 111 in 46- to 60-years-old group (*P* = 0.024), 12 out of 259 in 61- to 75-years-old group (*P* = 0.029) and 8 out of 111 in patients older than 75 years (*P* = 0.016). The distribution of gender, ethnicity, and education showed no difference between all groups. Patients were also divided into three groups, according to the time between implant surgery to PD or ischemia diagnosis, <5, 5–10, and >10 years. The first group showed more cases with metal inserts diagnosed with PD or ischemia (Figure S1C), the incidences were 12 out of 18 (66.67%, *P* = 0.002) and 13 out of 25 (52.00%, *P* = 0.044), respectively.

### The High PD-Occurrence after Orthopedic Surgeries with Metal Implants

In Table 2, we summarize the occurrence of PD after orthopedic surgeries with and without metal implants in the past 10 years. Since 2009, we counted 7500 subjects who underwent orthopedic surgeries without using metal implants and 15 000 subjects who had orthopedic surgeries with metal implantation. The occurrence of PD in surgeries without metal implants was 1.31%, whereas this ratio increased to 1.98%, in surgeries with metal implants; this difference reached the level of statistical significance (*X*^2^(1)=13.143, *P* < 0.001).

**Table 2.** Occurrence of Diagnosed PD after Orthopedic Surgeries without and with Metal Implants

Parameter	Cases without metal implants	Cases with metal implants
Total number	7500	15* *000
Age at surgery (years)		
<46	2403	5837
46–60	2218	5529
61–75	1954	2946
>75	425	688
Gender		
Male	3505	7829
Female	3995	7171
Ancestry		
Han race	7422	14* *857
Non-Han race	78	143
Diagnosed-PD	98	297
PD-ratio (%)	1.31	1.98
	*X* ^2^=13.143	*P *=* *0.000289*

* There is significant difference in the occurrence of diagnosed-PD between cases without and with mental implants groups.

**Figure 1.**
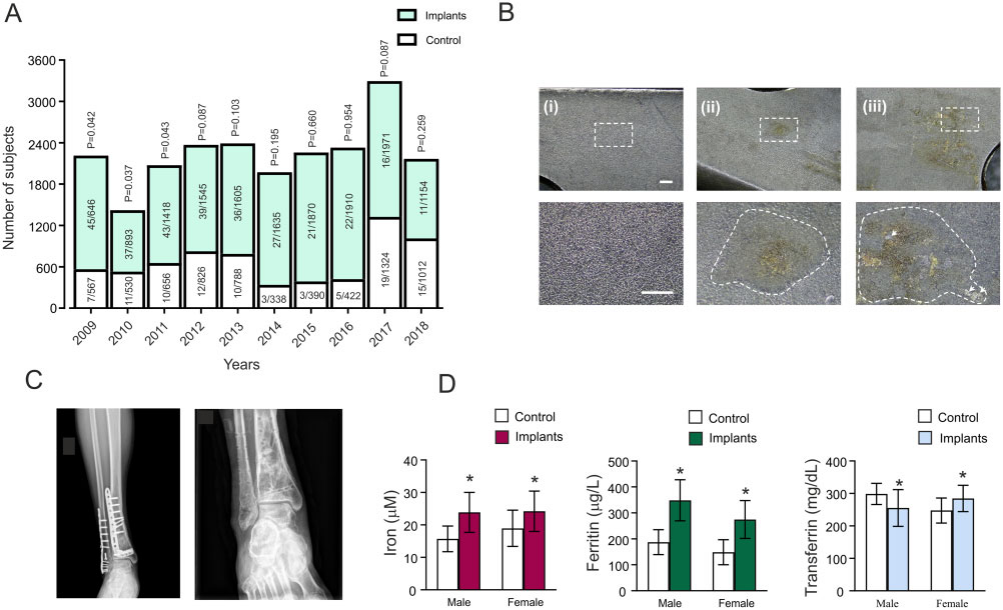
Occurrence of PD after Orthopedic Surgeries from 2009 to 2018. (**A**) The numbers of the subjects and PD diagnoses after orthopedic surgeries with and without metal implants. (**B**) The surface images (at 10× and 30× magnification) of the unused (i) and removed (ii), (iii) metal plates; (ii) shows the surface of the implant facing the bone; and (iii) the surface of the implant facing the muscle and fascia. Scale bar, 1 mm. Dotted lines show areas of corrosion. (**C**) X-ray images before and after removing metal implants. (**D**) The levels of serum iron, ferritin, and transferrin in the healthy control and subjects with metal implants, represented asmean ± SD, *n* = 30 in male group, *n* = 20 in female group. *P < 0.05—statistically significant difference from control group.

The yearly numbers of PD-diagnosed subjects and the total number of orthopedic surgeries with or without metal inserts are shown in Figure 1A. During the period between 2009 and 2011, we found significant difference between using metal implants surgeries group (implants group) and orthopedic surgeries without implants (control group). In 2009, 7 out of 567 subjects having orthopedic surgeries were diagnosed with PD, but the PD occurrence was 45 out of 1646 in metal inserts group (*X*^2^(1)=4.132, *P* = 0.042). In 2010, the PD occurrences in control and inserts groups were 11 out of 530 and 37 out of 893, respectively (*X*^2^(1)=4.363, *P* = 0.037). In 2011, the PD occurrence in these two groups was 10 out of 656 and 43 out of 1418 (*X*^2^(1)=4.096, *P* = 0.043). In the ensuing 7 years, there was no significant difference in the occurrence of PD between control and inserts groups.

The bleeding volume and anesthesia time were also compared between these two groups. As shown in Figure S2A, the bleeding volume during orthopedic surgeries without and with metal implants was 200 ± 4.01 mL (*n* = 7500) and 210 ± 3.08 mL (mean ± SEM, *n* = 15 000), there was no significant difference (*P* = 0.0544). The anesthesia times were 1.97 ± 0.04 h (mean ± SEM, *n* = 7500) and 2.07 ± 0.03 h (mean ± SEM, *n* = 15* *000) in surgeries without and with implants groups, respectively; again, there was no significant difference (*P* = 0.0556; Figure S2B).

We also compared the occurrence of PD in orthopedic surgeries with or without metal implants in upper limbs, spine, and lower limbs (Figure S2C). In upper limbs, the surgeries without implants included the dissection of the transverse carpal ligament and decompression of the median nerve, as well as suturing of wrist ligaments (*n* = 199). Surgeries with metal implants were represented by internal fixation of the distal radius fracture (*n* = 377). The PD occurrences were 1 out of 199 in the group without implants and 12 out of 377 in the group with implants (*X*^2^(1)=4.242, *P* = 0.039). In lumber spine, lumbar intervertebral disc nucleus pulposus resection and the posterior approach of bone graft fusion with screw fixation system for lumbar spine fracture, PD occurrence was 4 out of 512 and 23 out of 995 (*X*^2^(1)=4.499, *P* = 0.034). On lower limbs, for treating knee osteoarthritis, arthroscopic arthrodesis of the knee and total knee arthroplasty were performed. For these two surgeries, PD occurrence was, respectively, 6 out of 655 and 26 out of 1122 (*X*^2^(1)=4.592, *P* = 0.032).

### Increased Serum Iron and Ferritin in Subjects with Metal Implants

To detect the effects of implanted iron on the level of iron in body fluids, we collected the blood samples of healthy subjects and patients who experienced metal implants removal surgeries. These patients carried the implants for ∼1.5 years before removal. The representative X-ray images before and after removal surgeries are shown in Figure 1B. When examining the surface of the removed metal plates, we invariably found corrosions on the surface on these plates, on both sides adjoining either muscles or bones (Figure 1C). As shown in Figure 1D, serum iron in patients with implants was significantly higher when compared to the control group: in males, serum iron was 15.69 ± 3.96 µM in control groups and 23.85 ± 6.16 µM in implants group (*P* < 0.001, *n* = 30), in females: 18.93 ± 5.57 µM in control group and 24.19 ± 6.22 µM in implants group (*P* < 0.001, *n* = 20; Figure 1D). Serum ferritin in males was 187.20 ± 48.01 µg/L in control groups and 348.56 ± 79.11 µg/L in implants group (*P* < 0.001, *n* = 30), while in females ferritin was 148.46 ± 48.37 µg/L in control group and 274.41 ± 72.84 µg/L in implants group (*P* < 0.001, *n* = 20; Figure 1D). The level of transferrin in males was 298.46 ± 32.53 mg/dL in control groups and 255.20 ± 56.66 mg/dL in implants group (*P* < 0.001, *n* = 30), while in females transferrin was 247.37 ± 38.48 mg/dL in control group and 284.39 ± 40.70 mg/dL in implants group (*P* = 0.005, *n* = 20).

### Opposite Changes in DMT1 in Neurons and Glia in Response to Iron Overload

**Figure 2.**
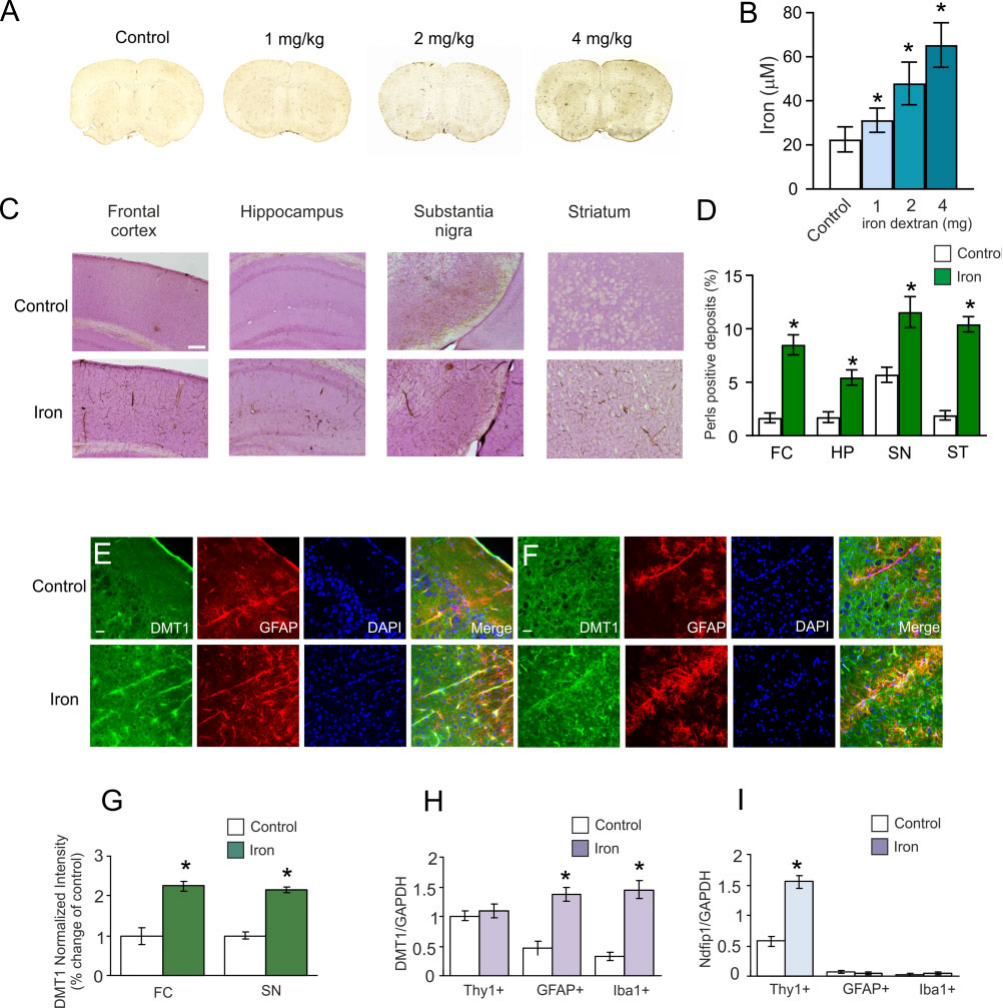
Iron Accumulation in the Brain and Expression of DMT1-specific mRNA. (**A**) Perl’s images of the whole brain slices obtained from animals treated with different concentrations of iron dextran (0, 1, 2, and 4 mg/kg/day) for 6 days. (**B**) The serum iron levels after injection with iron dextran (0, 1, 2, and 4 mg/kg/day) for 6 days, presented as mean ± SD, *n* = 6. *P < 0.05—statistically significant difference from control group. (**C**) Perl’s images of FC, HP, SN, and ST taken from control animals and animals subjected to 6 days injections of 2 mg/kg/day iron dextran. Scale bar, 500 µm. (**D**) The percentage of Perl’s stain represents mean ± SD, *n* = 6. *P < 0.05—statistically significant difference from control group. (**E** and **F**) Immunofluorescence images of DMT1 expressed in astrocytes around blood vessels after treatment with 2 mg/kg/day iron dextran. DMT1 (green), GFAP (red), DAPI (blue), and merged images were shown for FC (E) and SN (F). (**G**) The fluorescence intensity of DMT1 was normalized to control group and is presented as mean ± SD, *n* = 6. The cells we costained with antibodies against GFAP and DAPI. Scale bar, 25 µm. *P < 0.05, statistically significant difference from control group. (**H** and **I**) The relative mRNA expression ratio of DMT1 and Ndfip1. After treatment with 2 mg/kg/day iron dextran for 6 days, neurons (Thy1+), astrocytes (GFAP+), or microglial cells (Iba1+) were FACS sorted for qPCR measurement. The relative mRNA expression ratio of DMT1/GAPDH (H) and Ndfip1/GAPDH (I) is presented as mean ± SD, *n* = 6. *P < 0.05—statistically significant difference from control group.

To study the effects of iron in animals, we injected mice hypodermically with 200 µL iron dextran water solution which contained iron at 1, 2, or 4 mg/kg/day for 6 day, q.d. Iron accumulation was identified in the brain using Perl’s staining. Iron accumulation increased with an increase in the intravenously injected dose (Figure 2A). Increase in the iron concentration was detected in serum (Figure 2B); iron concentration levels (30–70 µM) were comparable to that detected in the serum of patients. When compared with the control group, iron dextran increased serum iron to 138.67 ± 24.44% (*P* = 0.023, *n* = 6) at 1 mg/kg, 212.89 ± 43.11% (*P* < 0.001, *n* = 6) at 2 mg/kg, and 290.22 ± 44.89% (*P* < 0.001, *n* = 6) at 4 mg/kg. Four cerebral regions, namely FC, HP, SN, and ST showed iron accumulation following 2 mg/kg iron injections (Figure 2C). When compared with the control group, the percentage of the positive stained area increased to 509.73 ± 56.09% (*P* < 0.001, *n* = 6) in FC, 315.08 ± 41.53% (*P* < 0.001, *n* = 6) in HP, 202.86 ± 25.53% (*P* < 0.001, *n* = 6) in SN and 546.33 ± 37.55% (*P* < 0.001, *n* = 6) in ST (Figure 2D). The expression of DMT1 in astrocytes around big vessels was also detected in FC and SN (Figure 2E–G). Iron increased the immunofluorescence intensity of DMT1 to 224.42 ± 30.41% (*P* < 0.001, *n* = 6) of the control group in FC (Figure 2G), and to 215.42 ± 19.04% (*P* < 0.001, *n* = 6) of the control group in SN (Figure 2G).

The Nedd4 family interacting protein 1 (Ndfip1) is known to regulate ubiquitination and degradation of DMT1 [19], being thus a leading factor defining transporter presence and operation. Expression of mRNA for DMT1 and Ndfip1 was determined in freshly isolated neurons, astrocytes, and microglial cells (Figure 2H and I). In FACS-sorted neurons, treatment with iron dextran did not significantly change mRNA expression of DMT1, but increased the mRNA level of Ndfip1 to 271.93 ± 47.27% (*P* < 0.001; *n* = 6) of the control group. Administration of iron dextran upregulated mRNA expression of DMT1 to 291.49 ± 62.54% (*P* < 0.001; *n* = 6) and 453.13 ± 114.82% (*P* < 0.001; *n* = 6) of control groups in astrocytes and microglial cells, respectively. However, iron treatment did not change mRNA expression of Ndfip1 in glial cells.

**Figure 3.**
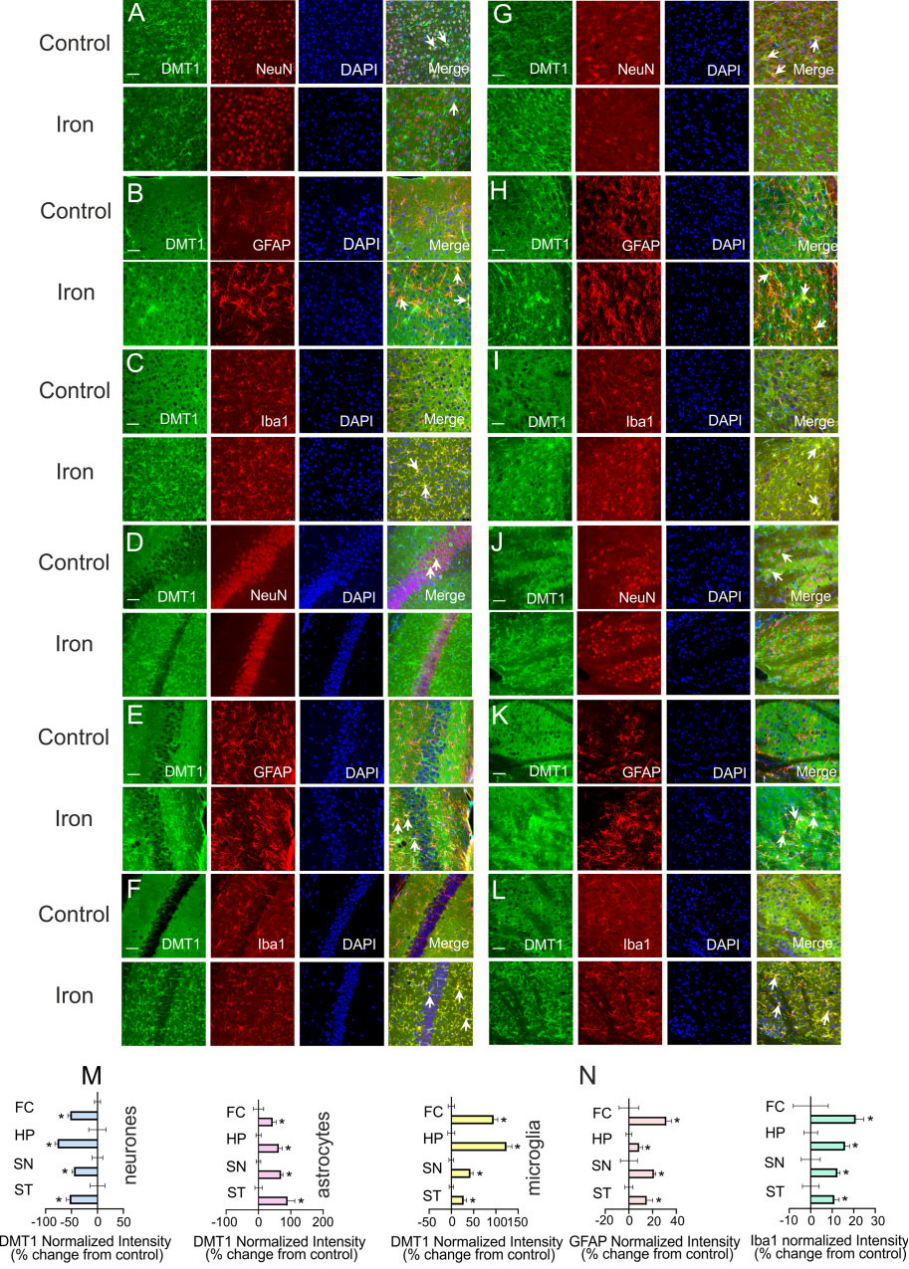
Protein Levels of DMT1 in Neurons and Glia in Four Cerebral Regions. **(A–L)** After treatment with 2 mg/kg/day iron dextran for 6 days, the images of DMT1 (green) costained with NeuN, GFAP, or Iba1 (red) and DAPI (blue) were visualized in FC (A–C), HP (D–F), SN (G–I), and ST (J–L). Scale bar, 50 µm. (**M**) The fluorescence intensity of DMT1 normalized to control for neurons, astrocytes, and microglia is presented as mean ± SD, *n* = 6. (**N**) Changes in fluorescent intensity of GFAP and Iba1 induced by treatment with 2 mg/kg/day iron dextran for 6 days. Data are presented as mean ± SD, *n* = 6. *P < 0.05—statistically significant difference from control group.

Treatment with iron dextran decreased fluorescent intensity of DMT1 immunostaining in neurons but increased it in astrocytes and microglia (Figure 3A–M). In FC, for example, when compared with control group, the fluorescence intensity of DMT1 in neurons decreased to 47.54 ± 11.95% (*P* < 0.001, *n* = 6), whereas it increased to 143.92 ± 24.96% (*P* = 0.021, *n* = 6) and to 195.30 ± 21.65% (*P* < 0.001, *n* = 6) in astrocytes and microglia (Figure 3M). Similar intensity changes of DMT1 were also observed in other three cerebral regions. The treatment with iron dextran decreased the fluorescence intensity of DMT1 in neurons to 22.93 ± 12.35% (*P* = 0.009, *n* = 6), 54.74 ± 9.55% (*P* < 0.001, *n* = 6), and 46.24 ± 15.87% (*P* = 0.006, *n* = 6) in HP, SN, and ST, respectively. In these three brain regions, the intensity of DMT1 was increased to 162.57 ± 27.21% (*P* < 0.001, *n* = 6), 169.54 ± 13.64% (*P* < 0.001, *n* = 6), and 189.94 ± 53.82% (*P* = 0.001, *n* = 6) in astrocytes, and it was elevated to 223.58 ± 29.76% (*P* < 0.001, *n* = 6), 142.82 ± 14.72% (*P* < 0.001, *n* = 6), and 127.62 ± 15.92% (*P* = 0.002, *n* = 6) in microglia, compared with control group (Figure 3M).

### Iron Accumulation Triggers Reactive Gliosis

Iron dextran significantly increased the fluorescence intensity of GFAP and Iba1 in four cerebral regions (Figure 3N). Compared with control group, the intensity of GFAP was significantly increased to 131.48 ± 11.24% (*P* = 0.008, *n* = 6), 108.71 ± 6.83% (*P* = 0.040, *n* = 6), 121.16 ± 3.67% (*P* = 0.017, *n* = 6), 115.03 ± 12.1% (*P* = 0.031, *n* = 6; Figure 3P), whereas the intensity of Iba1 was elevated to 120.91 ± 9.01% (*P* = 0.041, *n* = 6), 115.96 ± 5.04% (*P* = 0.002, *n* = 6), 112.42 ± 2.89% (*P* = 0.022, *n* = 6), 110.93 ± 5.46% (*P* = 0.035, *n* = 6) in FC, HP, SN, and ST, respectively (Figure 3N). Iron dextran increased the size of GFAP-profiles indicative of reactive astrogliosis. Compared with the control group, the average surface area of 3D restructured GFAP profiles was increased to 186.75 ± 65.17% (*P* = 0.025, *n* = 6) in FC, and to 174.11 ± 57.26% (*P* = 0.028, *n* = 6) in SN (Figure S3).

### Iron Accumulation Instigates Neuronal Apoptosis and Abnormal Behaviors

**Figure 4.**
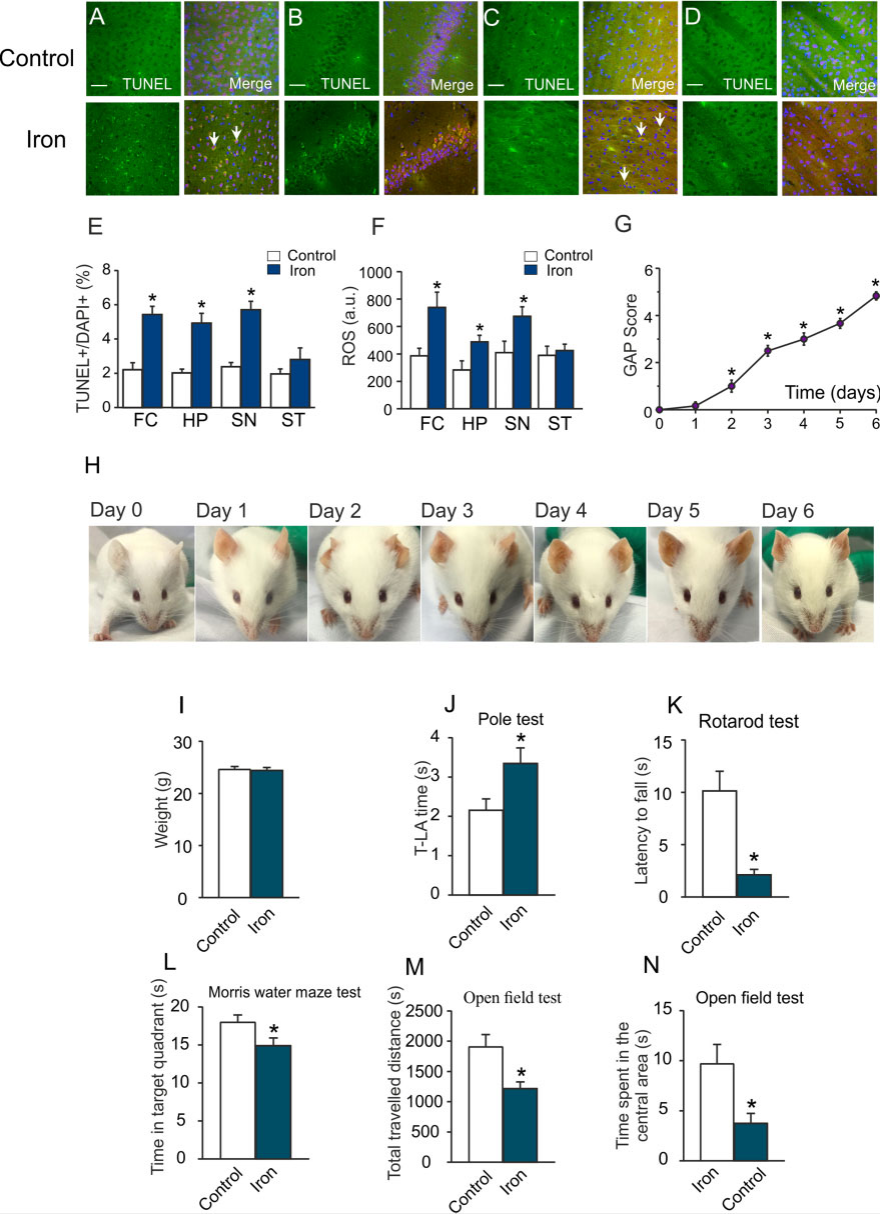
Effects of Excess Iron on Neuronal Apoptosis and Behavior. **(A–D)** After treatment with 2 mg/kg/day iron dextran for 6 days, TUNEL assays were applied to FC (A), HP (B), SN (C), and ST (D). Neurons were also stained with NeuN (red) and nuclei were labeled with DAPI (blue). (**E**) Ratios of TUNEL+/DAPI+ cells presented as mean ± SD, *n* = 6. Scale bar, 50 µm. *P < 0.05, statistically significant difference from control group. (**F**) ROS levels in four cerebral regions. Data presented as mean ± SD, *n* = 6. *P<0.05, statistically significant difference from control group. **(G–N)** After treatment with 2 mg/kg/day iron dextran for 6 days, the mice behaviors were tested. (G) GAP scores of animals treated with 2 mg/kg/day iron dextran from 0 to 6 days. Data represent mean ± SEM, *n* = 6. *P < 0.05, statistically significant difference from 0 day. (H) Appearances of mice treated with 2 mg/kg/day iron dextran from Day 0 to Day 6. (I) Weight of mice in control group and in a group treated with 2 mg/kg/day iron dextran for 6 days. (J) Movement time from the pole top to the floor (T-LA time). (K) The dwelling time on rotating bar. (L) Time spent in target quadrant in Morris water maze test. (M and N) The total traveled distance (M) and time spent in the central area (N). Data are presented as mean ± SD, *n* = 6. *P < 0.05, statistically significant difference from control group.

The ratio of TUNEL+/DAPI+ (total cells) was elevated to 243.45 ± 51.59% (*P* < 0.001, *n* = 6), 241.94 ± 65.70% (*P* < 0.001, *n* = 6), and 237.75 ± 47.99% (*P* < 0.001, *n* = 6) of control group in FC, HP, and SN; there was no significant difference between control and iron dextran groups in ST region (Figure 4A–E). Treatment with iron dextran significantly increased the level of ROS to 190.69 ± 69.59% (*P* = 0.017, *n* = 6) in FC, to 171.39 ± 38.58% (*P* = 0.026, *n* = 6) in HP, to 164.01 ± 40.22% (*P* = 0.031, *n* = 6) in SN, when compared to control group (Figure 4F). However, in ST iron dextran did not affect the level of ROS.

The general appearances of mice are shown in Figure 4G and H. The score of mice in absence of iron was 0, the GAP scores gradually increased during treatment with iron dextran, to 4.83 ± 0.42 (*n* = 6) at Day 6. Specifically, the eyes were glazed and sunken, and the color of ears darkened (Figure 4H). The average weight of mice was not affected by iron injection (Figure 4I). During the administration of iron dextran, the mice progressively developed a series of abnormal behaviors, such as restlessness, guarding, arched back, irregular breath, and segregation. In pole test, iron dextran prolonged the used T-LA time to 154.95 ± 43.99% of control group (*P* = 0.035, *n* = 6; Figure 4J). The dwell time in rotarod test was shortened by iron dextran to 21.31 ± 11.49% of control group (*P* = 0.002, *n* = 6; Figure 4K). Administration of iron dextran significantly decreased the time spent in target quadrant in Morris water maze test to 83.00 ± 13.06% of control group (*P* = 0.029, *n* = 6; Figures 4L and S4). In open field test, the travel distance and time spent in central area were decreased to 63.98 ± 13.84% (*P* = 0.010, *n* = 6) and 38.97 ± 23.96% (*P* = 0.017, *n* = 6), compared with control group (Figure 4M and N).

## Discussion

**Figure 5.**
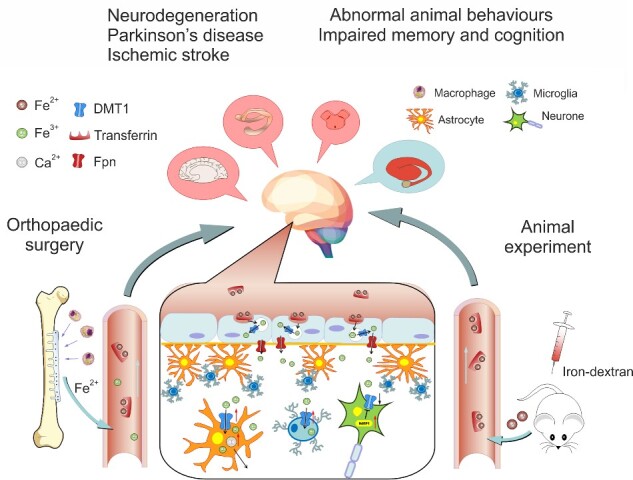
Schematic Rendition of the Effects of Excess Iron on Neural Cells. After inserting metal implants in orthopedic surgery, or after injection of iron-dextran to experimental animals the ionized Fe^2+^/Fe^3+^ enters the bloodstream. Through transportation of transferrin and DMT1, Fe^2+^/Fe^3+^ crosses BBB and blood–cerebrospinal barrier and accumulates in the brain. Facing the attack of excess iron, glial cells (astrocytes and microglia) act as protectors by increasing the uptake of ferrous by upregulating DMT1, the main iron transporter. At the same time, neurons restrict the influx of iron by degrading the DMT1 by upregulation of Ndfip1. This phenomenon has been observed in several cerebral regions, including FC, HP, SN, and ST. Pathological increase in iron levels is linked to an increased ROS production and neuronal apoptosis. Neuronal death and reactive changes in glia may instigate abnormal animal behaviors, impair memory, and cognition and contribute to the development of neurodegenerative diseases.

Iron is a necessary element in the human body, the iron deficiency is harmful for development and health, but the excessive iron can also trigger pathology. According to our study (as summarized in Figure 5), the insertion of iron-based metal implants in the orthopedic surgery may, in long-term, affect the nervous system and, as we found, may increase the risk of PD and ischemic stroke. Grafting of iron implants increased probability of PD or ischemia during first 5 years after intervention. Here we focused on the link between iron implants and PD. We will further investigate effects of iron on the brain ischemia in our future research, concentrating, in particular, on possible action of iron on vascular endothelial cells. The confirmed age for patients diagnosed with PD and metal implants tends to exceed 60 years, which agrees with the average onset age of this disease. The occurrence of PD was significantly increased in the metal implants group compared with patients with orthopedic surgery not associated with metal implanting. Furthermore, the patients undergoing surgery for removing metal implants had higher levels of serum iron and ferritin.

We found clear signs of corrosion in the metal implants removed from patients (Figure 1C). The increased serum iron and ferritin levels in subjects bearing metal implants reflect an increased level of iron in the body. Ferritin levels indicate iron reserve; physiological ferritin levels vary depending on gender, age, and other factors.^22^ Serum ferritin levels exceeding 300 μg/L in males and 200 μg/L in females are considered abnormal.^22^^,^^23^ Serum ferritin levels detected in patients with implant removal surgery were 348.56 ± 79.11 µg/L and 274.41 ± 72.84 µg/L, in males and females respectively (see Figure 1D), indicating iron overload. We found no difference in the rates of PD occurrence between patients with surgeries in different locations, suggesting the role of implant presence and not for its location.

To further analyze mechanisms of iron action on CNS, we studied the effects of hypodermic injections of iron dextran on the expression of major iron transporter DMT1 in different types of neural cells. We found that treatment with iron changed protein expression of DMT1 in neurons and glial cells in the opposite direction. In all four brain regions under analysis (FC, HP, SN, and ST), iron decreased neuronal DMT1 protein content, whereas protein levels of DMT1 were increased in astrocytes and in microglial cells. We contemplated that this phenomenon reflects a neuroprotective mechanism activated by iron attack. As summarised in Figure 5, glial cells increase the uptake of ferrous by upregulating DMT1 thus reducing iron load and protecting nervous tissue. Similar DMT1 upregulation was identified in astrocyte perivascular endfeet forming the *glia limitans perivscularis*. Our findings in situ confirm previous observations of upregulation of DMT1 in iron-treated astrocytes in culture.^24^ In contrast to astrocytes, neurons restrict the influx of iron by decreasing DMT1 presence. Decrease in protein level of DMT1 in neurons was not due to the downregulation of relevant gene transcription, but resulted from increased degradation of DMT1 by Ndfip1, an adaptor protein for Nedd4 family of ubiquitin ligases,^25^^,^^26^ because neuronal mRNA of Ndfip1 was significantly and selectively increased by iron. Chronic treatment with iron was shown to increase the expression of Ndfip 1 in MES23.5 cells,^27^ although Ndfip1 was not detected in glial cells.^28^ Our results support the notion of absence of Ndfip1 in glia with or without iron overload. In addition, excessive iron triggers reactive gliosis (as indicated by an increase in expression of GFAP and Iba1 and an increase in the size of GFAP-positive astrocytic profiles), which may contribute to overall glia-dependent neuroprotection.^29^ Hence, facing the invasion of excess iron, glial cells mount gliotic response and protect neurons by taking up excess of extracellular iron.

Chronic treatment with iron elevated ROS production in the FC, HP, and SN, but not in the ST. Neuronal apoptosis induced by iron and mediated by ROS was similarly identified in these three regions. Behavioral tests demonstrated that treatment with iron evoked anxiety behaviors and resulted in cognitive and movement dysfunction. Dopaminergic neurons are known to be particularly vulnerable to oxidative damage^30^ and indeed this was reflected by the difference in apoptosis level between SN and ST (Figure 4F). Early and prominent neuronal apoptosis observed in SN when compared to the ST may explain the link to clinical symptoms of PD associated with relatively hyperactive acetylcholine function. Following treatment with iron dextran cerebral, depositions of iron were gradually increased and mice developed abnormal appearance, poor coordination, locomotion, declined cognition, and increased anxiety-like behaviours.

In this study, because of the limitation of research time and recorded information, we could not further compare the PD occurrence in patients with metal implants according to the different metal components of implants, the plates coating, their weight, or production origin. The PD occurrence was larger during 2009–2011 period (Figure 1A). That may result from the higher usage of the metal implants without coating, or simply reflect the long incubation period of PD. Our studies do not exclude other metal components which may also increase the risk for the neurodegenerative diseases, because some metals can also be released into the body after using metal implants.^31–33^ All these particularities merit future studies. According to our data, the levels of serum iron and ferritin should be monitored in subjects having metal implants.

In conclusion, neuronal apoptosis and reactive gliosis accompany iron overload which promotes neuropathology. The excessive iron should be of special concern and the patients taking iron-based metal implants in orthopedic surgeries have to be considered at risk of the neurodegenerative diseases.

## Supplementary Material

Supplementary material is available at the *APS Function* online.

## Funding

This study was supported by Grant No. 81871852 to B.L. from the National Natural Science Foundation of China, Grant No. XLYC1807137 to B.L. from LiaoNing Revitalization Talents Program, Grant No. 20151098 to B.L. from the Scientific Research Foundation for Overseas Scholars of the Education Ministry of China, and Grant No. 202078 to B.L. from Liaoning BaiQianWan Talents Program. Grant No. 81200935 to M.X. from the National Natural Science Foundation of China, Grant No. 20170541030 to M.X. from the Natural Science Foundation of Liaoning Province. Grant No. 81671862 and No. 81871529 to D.G. from the National Natural Science Foundation of China.

## Authors’ Contributions

M.X., A.V., D.G., and B.L. designed and supervised the study; M.X., Shuai Li (S.L.), C.D., Be.C., Bi.C., M.J., Y.Z., and W.G. collected the clinic messages and analyzed the relevant data; Sha.L., G.W., X.Z., D.Z., X.L., and M.Z. performed the experiments in vivo and analyzed the data; B.L. and A.V. wrote the manuscript.

## Conflict of Interest Statement

The authors have no conflicts of interest to disclose.

## Supplementary Material

zqab003_Supplementary_Data
