# Supplementary material for: Iatrogenic Iron Promotes Neurodegeneration and Activates Self-Protection of Neural Cells against Exogenous Iron Attacks
Source: Function (Oxf). 2021 Jan 12;2(2):zqab003. doi: 10.1093/function/zqab003 (PMC8788796; doi:10.1093/function/zqab003)
Supplement: zqab003_Supplementary_Data [file zqab003_supplementary_data.pdf]

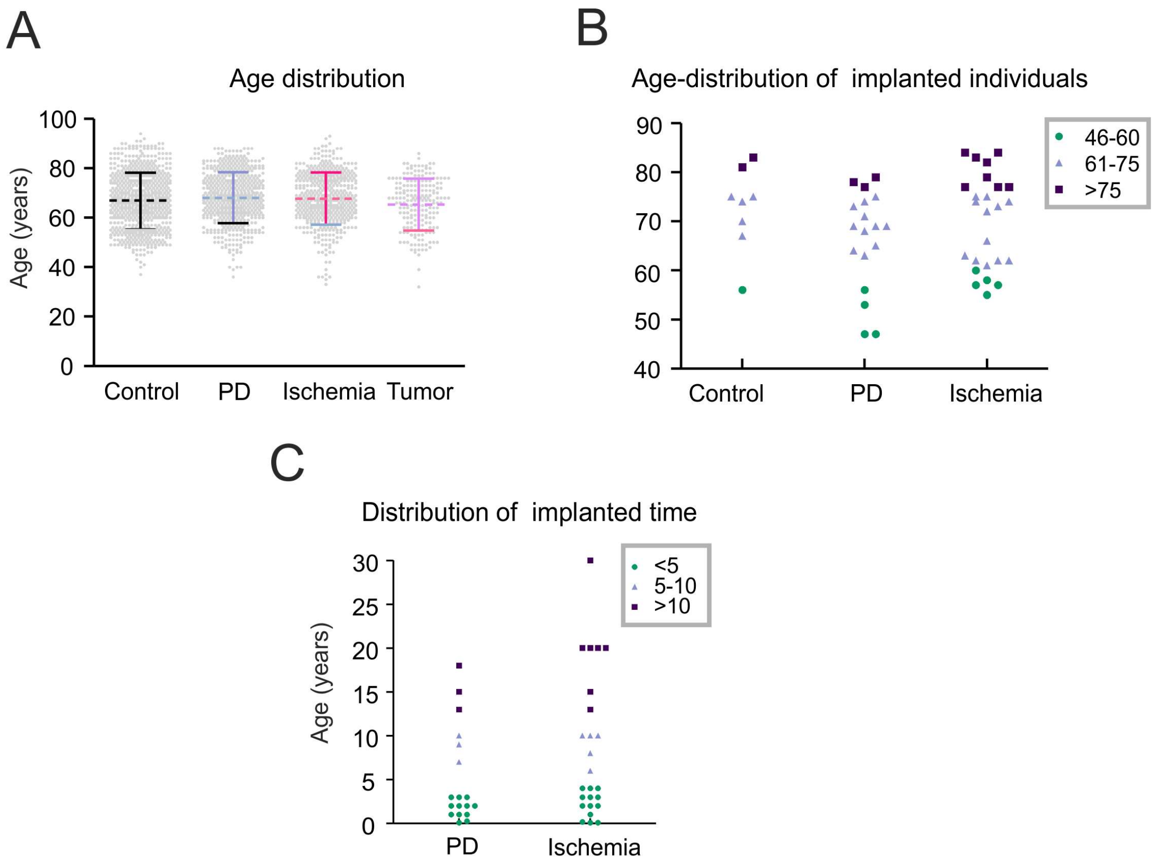

Supplementary Figure 1. The scatter diagram of implanted age and time in cases of PD and ischemia.

(A) Scatter diagram of average age in healthy subjects, PD, ischemia and tumour patients. (**B**) The age distribution of the individuals having metal implants. (**C**) The time distribution between metal implanted surgeries and the diagnosed time.

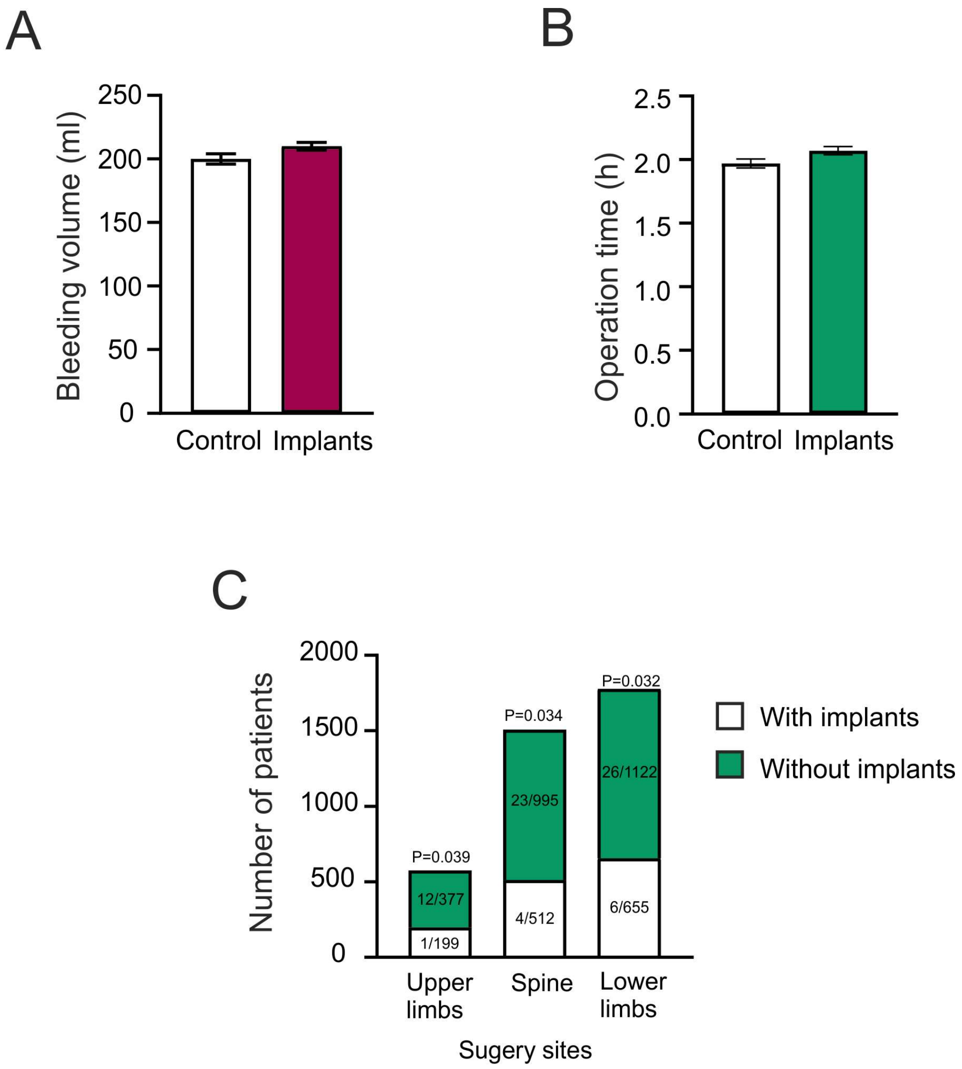

Supplementary Figure 2. The comparison of bleeding volume, surgery time and incidence of PD following surgeries at different locations.

(**A**) The bleeding volume of orthopaedic surgeries with and without using metal implants. (**B**) The anesthesia time during the orthopaedic operations with and without using metal implants. Data are presented as mean ± SEM, n=7500 in control group, n=15000 in implants group. (**C**) The numbers of the subjects and PD diagnoses after orthopaedic surgeries with and without using metal implants, respectively in upper limbs, spine and lower limbs.

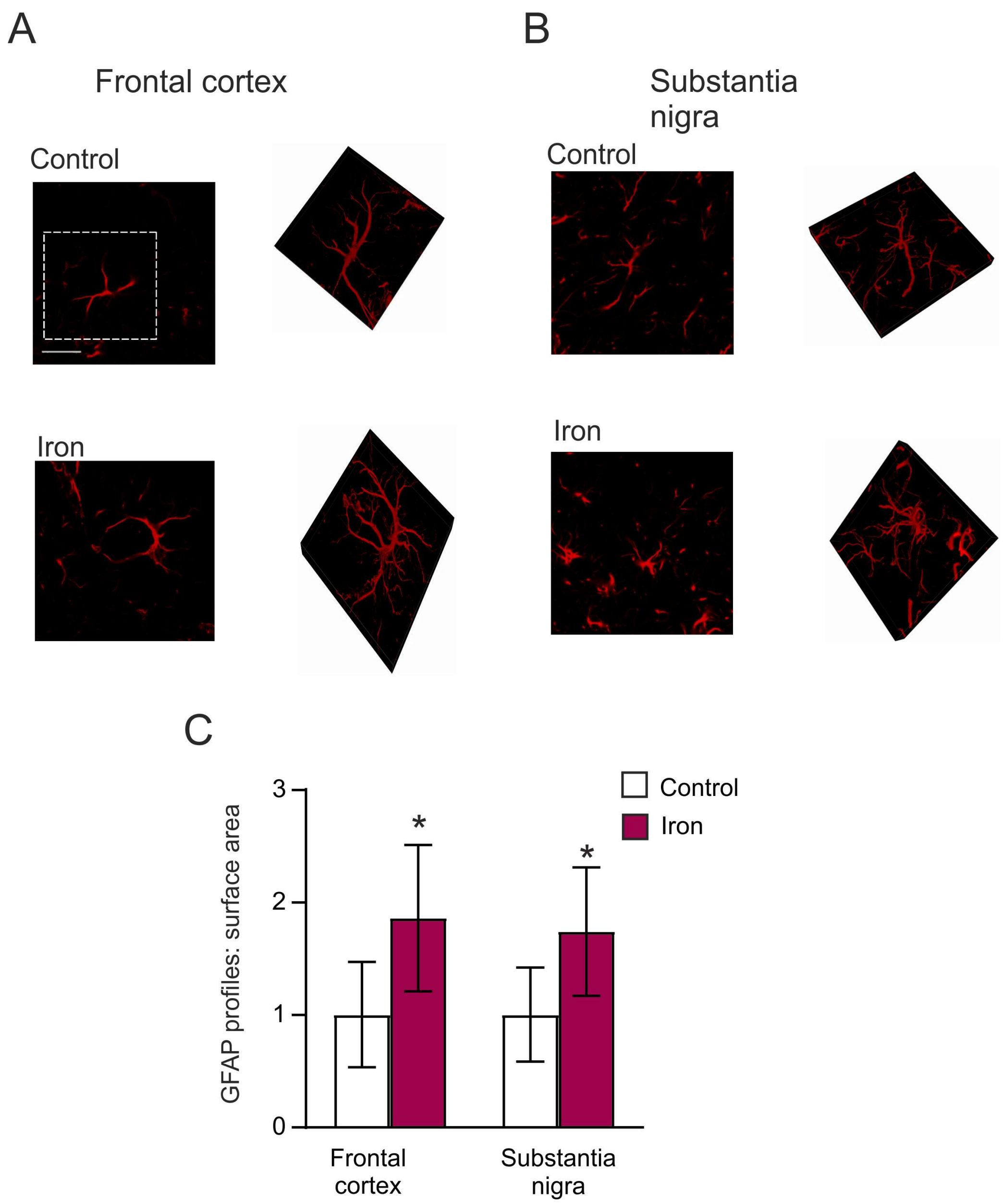

Supplementary Figure 3. 3D-images of GFAP immunofluorescence in frontal cortex and substantia nigra.

After treatment with 2 mg/kg/day iron dextran for 6 days, 3D-images of GFAP were constructed for FC (A) and SN (B). Scale bar, 20 μm. (C) The suface area of 3D reconstructed GFAP profiles was normalized to the control group. Data are presented as mean ± SD, n = 6. *p<0.05, denotes statistically significant difference from the controls.

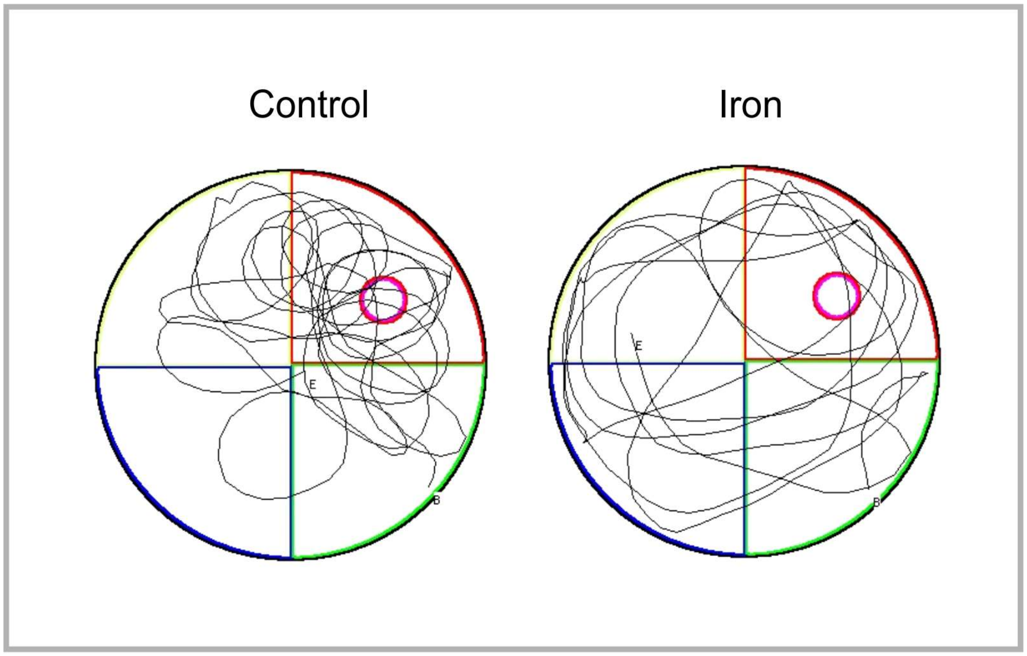

Supplementary Figure 4. The path diagram of mice in Morris water maze.

The representative path diagrams of mice with or without administration of iron dextran.

Supplementary Table 1. Metal implants distribution of Parkinson's disease cases, cerebral ischemia cases and healthy controls.

Feature	Healthy Control	PD	Cerebra Ischemia
**Total/Inserts number**	**700/8**	**500/18**	**500/25**
**Age at diagnosis (years)**			
<46	9/0	41/0	19/0
46-60	**199/1**	**177/4**	**111/5**
		P=0.192	**P=0.024 ***
61-75	**322/5**	**244/11**	**259/12**
		**P=0.036 ***	**P=0.029 ***
>75	**170/2**	**38/3**	**111/8**
		**P=0.043 ***	**P=0.016 ***
**Gender**			
Male	4 (50.00%)	11 (61.11%)	13 (52.00%)
Female	4 (50.00%)	7 (38.89%)	12 (48.00%)
		P=0.683	P=1.000
**Ancestry**			
Han ethical origin	7 (87.50%)	17 (94.44%)	25 (100.00%)
non-Han ethical origin	1 (12.50%)	1 (5.56%)	0 (0.00%)
		P=0.529	P=0.242
**Education level**			
Primary and secondary education	5	10	16
Skilled vocation and High school education	2	4	5
University and postgraduate education	1	4	4
		P=1.000	P=1.000
**Time of metal inserts (From surgery to diagnosis)**			
<5 years		**12 (66.67%)**	**13 (52.00%)**
5-10 years		**3 (16.67%)**	**5 (20.00%)**
>10 years		**3 (16.67%)**	**7 (28.00%)**
